# Divisive Gain Modulation with Dynamic Stimuli in Integrate-and-Fire Neurons

**DOI:** 10.1371/journal.pcbi.1000365

**Published:** 2009-04-24

**Authors:** Cheng Ly, Brent Doiron

**Affiliations:** Department of Mathematics, University of Pittsburgh, Pittsburgh, Pennsylvania, United States of America; Université Paris Descartes, Centre National de la Recherche Scientifique, France

## Abstract

The modulation of the sensitivity, or gain, of neural responses to input is an important component of neural computation. It has been shown that *divisive* gain modulation of neural responses can result from a stochastic shunting from balanced (mixed excitation and inhibition) background activity. This gain control scheme was developed and explored with static inputs, where the membrane and spike train statistics were stationary in time. However, input statistics, such as the firing rates of pre-synaptic neurons, are often dynamic, varying on timescales comparable to typical membrane time constants. Using a population density approach for integrate-and-fire neurons with dynamic and temporally rich inputs, we find that the same fluctuation-induced divisive gain modulation is operative for dynamic inputs driving nonequilibrium responses. Moreover, the degree of divisive scaling of the dynamic response is quantitatively the same as the steady-state responses—thus, gain modulation via balanced conductance fluctuations generalizes in a straight-forward way to a dynamic setting.

## Author Summary

Many neural computations, including sensory and motor processing, require neurons to control their sensitivity (often termed ‘gain’) to stimuli. One common form of gain manipulation is divisive gain control, where the neural response to a specific stimulus is simply scaled by a constant. Most previous theoretical and experimental work on divisive gain control have assumed input statistics to be constant in time. However, realistic inputs can be highly time-varying, often with time-varying statistics, and divisive gain control remains to be extended to these cases. A widespread mechanism for divisive gain control for static inputs is through an increase in stimulus independent membrane fluctuations. We address the question of whether this divisive gain control scheme is indeed operative for time-varying inputs. Using simplified spiking neuron models, we employ accurate theoretical methods to estimate the dynamic neural response. We find that gain control via membrane fluctuations does indeed extend to the time-varying regime, and moreover, the degree of divisive scaling does not depend on the timescales of the driving input. This significantly increases the relevance of this form of divisive gain control for neural computations where input statistics change in time, as expected during normal sensory and motor behavior.

## Introduction

Gain modulation (or gain control) is an adjustment of the input-output response of neurons, and is widely observed during neural processing [1]. Gaze direction sets the response gain in primary visual [2], posterior parietal cortex [3], and auditory brainstem [4]. In specific species, gain control mechanisms produce an invariance of receptive field properties [5] and orientation selectivity [6] to changes in overall stimulus contrast. Higher cognitive processes, such as attention, modulate the response gain of cells in primary visual cortex [7], as well as in V4 [8]. Finally, it has recently been shown that gain control schemes are needed to control behavior in invertebrates [9]. Despite the clear importance of gain control in a variety of neural computations, the biophysical mechanisms that support specific gain control mechanisms have been elusive [10]–[20].

Noise induced phenomena in nonlinear systems are a rich avenue of study [21], with recent interest on the impact of fluctuations on excitable systems, such as neurons [22]. Chance et al., Doiron et al., and Hô & Destexhe [11],[16],[23] all report that an increase in the fluctuations of background conductance inputs results in a decrease of the overall gain of the transfer between a static driving input and the mean output firing rate. In particular, if the balance between background excitation and inhibition is carefully controlled [11], then the gain control is purely divisive (or multiplicative). This means that an increase in conductance fluctuations acts to scale the transfer function over a large range of input by a simple constant multiplier (<1). Related work has further explored the impact of fluctuations on spike response [13]–[15],[20],[24],[25], with a the manipulation of the neural transfer function by background fluctuations being a central focus.

These studies address the gain control of a transfer function where the signal is either static or statistically stationary and the neural output is the time averaged firing rate. However, many neural coding tasks involve the processing of time-varying, high frequnecy stimuli. In these situations neural response are often transient, and a quasi-static approximation of input-output transfer fails to capture the actual spike response. For example, in the rodent vibrissa sensory [26], auditory [27]–[29], and electrosensory systems [30] stimuli and responses modulate on the order of a few milliseconds, i.e., on the order of, or even faster, than typical membrane time constants of neurons. Even in the visual system, where the relevant timescales of natural scenes are much slower, the response precision of thalamic neurons is at the millisecond level, and standard static transfer function analysis fails to capture neural response [31], yet contrast induced gain control persists [32]. In this study, we address the question of whether the fluctuation induced gain control mechanism explored for static transfer [11],[16],[23] can be operative for dynamic stimuli as well.

Any theoretical treatment of this problem requires 1) a framework accurately capturing the time varying spike response owing to time varying input statistics (e.g. temporally inhomogeneous input and output statistics), and 2) sufficient biophysical detail to incorporate conductance based synaptic inputs within spike creation. A useful tool for incorporating these two features into neuron models is the population density method [33]–[39]. In particular, Nykamp & Tranchina [35] have developed a simple one-dimensional population density method of conductance based leaky integrate-and-fire models (LIF). The one-dimensional version of the population density method allow us to easily study the firing rate responses to dynamic stimuli in the conductance based formalism of Chance et al. [11]. Minor differences in our proposed model and their dynamic clamp experiments to mimic conductance based inputs are presented in the discussion section. We first show that divisive gain modulation of the steady-state responses only hold for low output firing rates, in particular, where neurons are in the classical subthreshold regime. Second, when restricted to this regime we find the transient responses to dynamic stimuli, which can differ greatly from the quasi-static equilibrium response, also exhibit divisive gain modulation via fluctuation background conductances with the same scaling factor as computed in the static case. Thus, the divisive gain modulation proposed by Chance et al., Doiron et al., and Hô & Destexhe [11],[16],[23] generalizes to the dynamic situation in a very natural way.

## Methods

### Integrate-and-fire neuron

We consider a leaky integrate-and-fire neuron (LIF) driven by a pre-synaptic population of excitatory (e) and inhibitory (i) cells. The neuron's voltage change is given by a random differential equation:
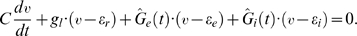
(1)Dividing by the leakage conductance 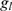 yields:
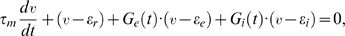
(2)

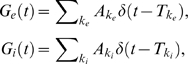
where 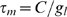 is the membrane time constant, 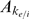 is the random size (for simplicity, chosen from the same distribution) of the excitatory/inhibitory 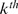 synaptic event. The arrival times 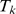 of both excitatory and inhibitory synaptic inputs are governed by modulated Poisson processes with mean rates 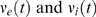, respectively. Throughout, 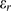 is the resting membrane voltage, 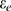 is the excitatory while 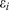 is the inhibitory reversal potential. When the neuron's voltage crosses 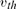, a spike is recorded and the neuron enters a refractory period for a fixed time of 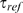, after which, its voltage is reset to 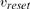. Consequently, the neuron's voltage 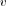 varies between 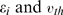 (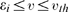). Throughout this paper, we will set 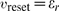, 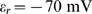, 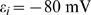, 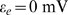, 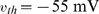, 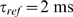, and 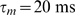 in accordance with estimates from experimental measures. We choose the average value of the random variables 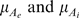 so that the neuron's voltage change (from 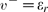) is ±0.5 mV [40]. The random variable 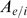 has a parabolic distribution function with finite support: 
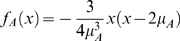
 for 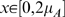 and 0 otherwise. It is convenient to define a new random variable 
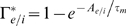
, because upon receiving an excitatory synaptic event, the neuron's voltage will increase by 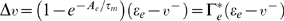 (see Nykamp & Tranchina [35] for a derivation). The neuron's voltage will decrease in a similar way upon receiving an inhibitory event: 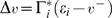. Thus, 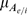 satisfy: 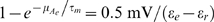 and 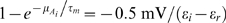.

We decompose the pre-synaptic input into a time-inhomogeneous ‘driver’ term 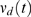, and time-homogeneous background terms 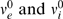:
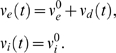
(3)The background synaptic activity is *balanced* [11],[40], namely 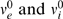 are chosen so that, in the absence of the driver input (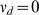), the random target voltage will have mean equal to the resting potential: 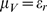. This will be true if:
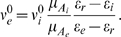
(4)


Of interest are the output threshold crossing times, and we estimate response statistics by combining the responses from 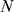 trials where the arrival times 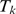 are statistically independent across trials yet share the same generating intensities 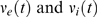. The instantaneous firing rate of the neuron is defined as
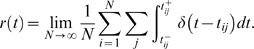
(5)where 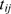 is the 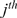 threshold crossing recorded during trial 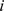. Throughout the paper we are interested in the relationship between the driver 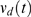 and the response 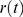, and specifically how the balanced activity 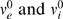 can modulate the relationship.

**Figure 1:**
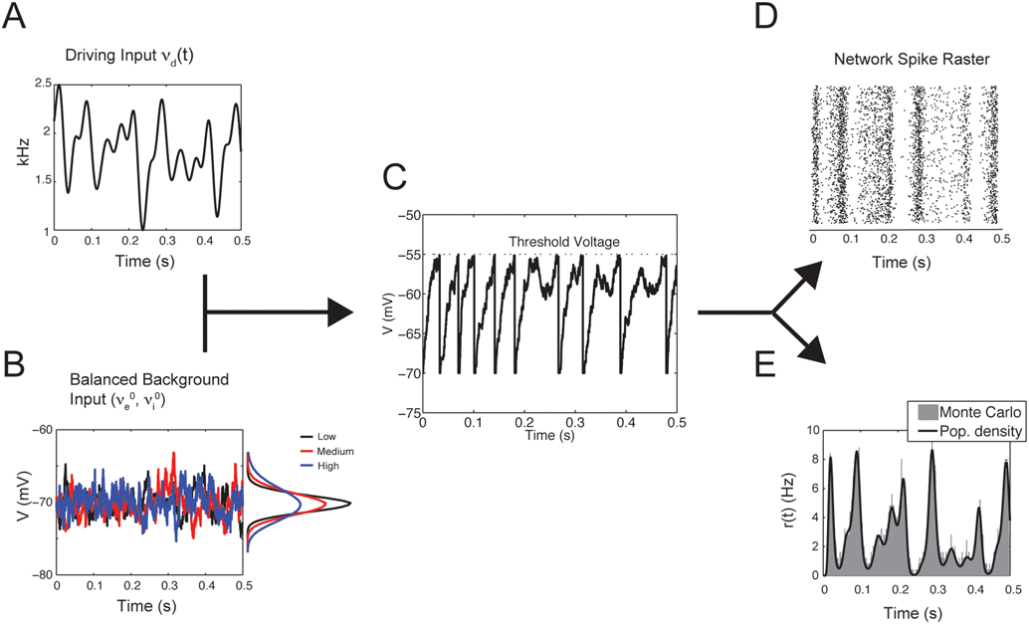
Input-output schematic for population of LIF neurons with a combination of driving input and balanced background fluctuations. (A) Excitatory driving input with rate 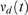. (B) Balanced fluctuating background inputs with rates 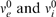. For illustrative purposes the evolution of 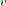 is shown when 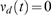; three intensities of background input are shown. (C) Sample realization of the LIF dynamic. (D) Raster plots of the output spikes are shown (Monte Carlo). (E) The output firing rate 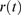, computed by the population density method (see Equations (6)–(8)), is a fast and efficient method for capturing the output firing rate. It matches the average firing rate of 100,000 random LIF neurons computed by Monte Carlo simulation.

**Figure 2:**
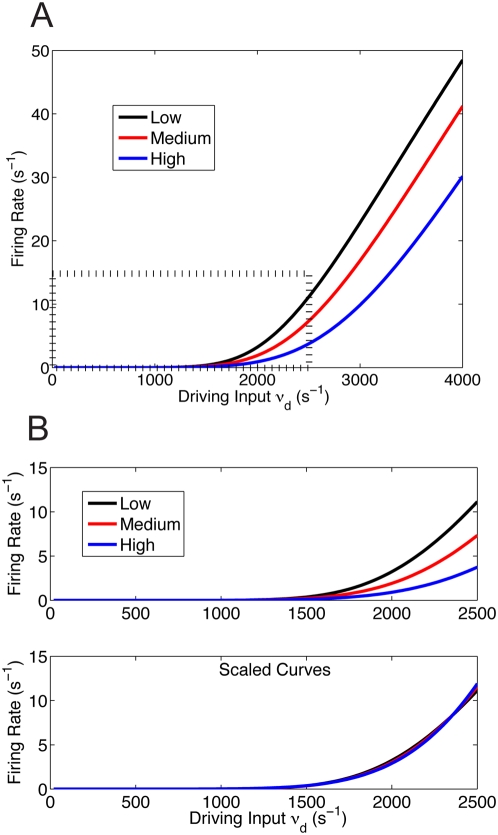
Gain modulation in the steady state. (A) The response curve 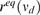 for different levels of balanced background activity (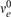 is determined once 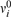 is specified, see Equation (4)). For high output firing rates outside of the boxed region, the slopes of all of the response curves are almost equal. (B) Top Panel: zoomed region of (A). Bottom Panel: a least squares fit was used to find the scalar 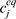 (see Equation (11)), and we plot the scaled response 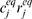. Here, 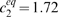 and 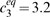.

Figure 1 is a schematic diagram of the representative leaky integrate-and-fire neuron from the population receiving the combination of driver and balanced inputs. For the sake of exposition, we focus on three different intensities of balanced background inputs: 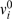 to be 1100 s^−1^, 1400 s^−1^, and 1900 s^−1^ (with corresponding 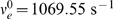, 1361.24 s^−1^, and 1847.40 s^−1^, respectively), which we respectively label *low* (black), *medium* (red), and *high* (blue). Chance et al. [11] modified the background level by various rate factors and labeled the regimes 1X, 2X, etc., which is slightly different than our convention of *low*, *medium*, and *high*. However, the resulting steady-state input/output curves (Fig. 2) are similar to those in Chance et al. [11]. Also, our results below hold equally well for many other sets of balanced background activity. For a particular background intensity (low in this case) with random excitatory drive 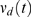, the output is random (see spike raster plots). As the balanced background activity is increased, the variability in the voltage also increases (Fig. 1B).

### Population density approach

A Monte Carlo simulation of Equation (2) would be computationally expensive to ensure an accurate result. In many studies only qualitative effects are reported, and thus quantitative accuracy is not at a premium. However, in our study the accuracy demands are large, as we will quantitatively compare the time dependent 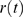 for various levels of background intensities. To overcome the errors inherent in finite data from Monte Carlo simulations we use population density methods [35], known to give very accurate estimates of 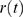 (Fig. 1E) for the idealized neural models described by Equations (2)–(5).

In the population density method, neurons with similar biophysical properties are grouped together, and the evolution of a density function 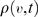 is considered. In brief, 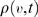 describes the voltage probability density over many statistically independent neurons. Integrating the density over a region in state space gives the probability that a neuron randomly chosen from the population will be in that region of state space:
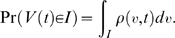
(6)


Let 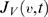 denote the probability current; a signed quantity with the convention that positive/negative 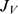 is the probability per unit time of crossing 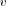 from below/above. The evolution of 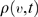 is governed by a continuity equation [35]:
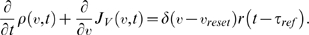
(7)We separate the probability current into three distinct terms:
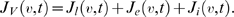
The first term, 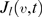 represents the deterministic leak to rest in the absence of synaptic events. The second and third terms, 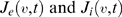, model the excitatory and inhibitory synaptic input driving the population. Mathematically we have:
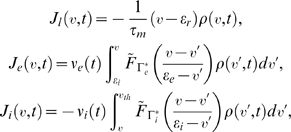
where 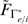 is the complementary cumulative distribution function: 
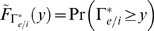
, for 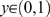. With the chosen distribution for 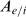, the functions above are (setting 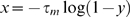):
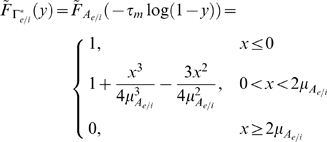
Finally, the instantaneous firing rate, 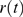, is the flux of probability current through 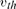 from below:
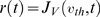


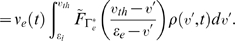
(8)The firing dynamics are implemented by an absorbing boundary condition at spike threshold, 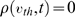, and the source term 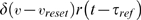 in Equation (7), modeling membrane reset after a refractory delay. The population average firing rate 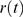 by the population density method (see Eqs (6)–(8)) is a computationally efficient way of capturing 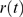, compared to computationally expensive Monte Carlo simulations.

### Divisive gain modulation

Gain modulation is typically studied in the equilibrium regime [10],[11],[16], where the driver input is constant in time 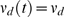 and the response 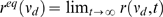 denotes the equilibrium firing rate as a function of input. A *divisive* gain modulation for 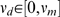 satisfies
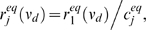
(9)where 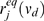 is the response of the population with some background activity ‘j’ (for our purposes j = 1 is low, j = 2 is medium, and j = 3 is high background activity) and 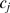 is a scalar. To measure the divisive gain modulation of a response 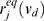 we fit the scaled response curves to 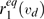 (*low*) by minimizing the mean-squared error 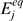:
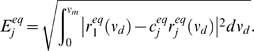
(10)Finding the 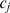 that minimizes 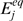 is easily obtained by orthogonally projecting onto the subspace spanned by 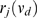:
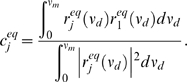
(11)Because we want the largest possible range for divisive gain modulation, we steadily increase the maximum of 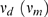 until the error in (10) becomes significant (i.e., the scaled curves no longer lie on top of each other). Let that maximum 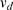 value be 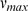.

The nonequilbirum response 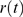 to a time-varying input 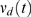 is given by Equation (8). We extend divisive gain modulation to the nonequilbrium setting, with the analogous description:
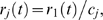
(12)

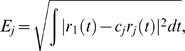
(13)

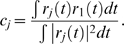
(14)The degree of divisive gain modulation in the nonequilibrium setting is determined by how well the time-varying responses scale with one another over a range of 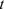. Thus, the scalar 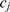 is now computed from integrals over 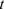, compared to integrals over 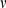 for the equilibrium case (11).

## Results

### Divisive gain modulation in the steady state for subthreshold firing rates

We compute the equilibrium input/output relationship, 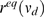, using the same framework as Chance et al. [11] (section: Integrate-and-fire Neuron), in hopes of first reproducing their results. Using balanced excitation and inhibition to mimic background synaptic activity, we compute 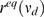 for different fixed values of the driving input 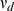 (Fig. 2). To obtain computationally accurate results in reasonable time we employ population density methods (section: Population Density Approach) to estimate the response 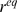. Divisive gain modulation via increases of the background rates 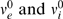 occurs in the low firing rate region (boxed region of Fig. 2A). In this regime the neuron response is dominated by input fluctuations rather than any intrinsic spike rhythmicity, thereby replicating the high variability observed in the spike responses in cortical networks [40]. Throughout we refer to this as the *fluctuation driven regime*. In the fluctuation dominated regime the responses can be scaled, in the sense of Equations (10)–(11), to nearly quantitatively match one another (Fig. 2B). This rescaling of the response by background fluctuations qualitatively matches the results presented in [11].

In contrast, for very high output firing rates divisive gain modulation does not occur. The responses 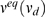 are nearly linear with very similar slopes (Fig. 2A), showing only a background activity induced translation of the response (often termed subtractive gain modulation [10]). This region corresponds to a regime where input fluctuations have limited impact and the neuron response is predominately determined by the mean value of the input rates, and we refer to this as *drift dominated regime*. Fluctuation induced divisive gain control restricted to low firing rates is consistent with [16], where simulations of a large-scale compartmental neuron model were used. The insensitively of 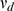 to input fluctuations at large 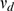 has also been recorded in pyramidal cells and fast-spiking interneurons [41],[42]. However, the exact 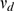 where gain manipulation changes from divisive to subtractive (as 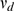 increases) is difficult to compute and is often model specific [14]. Indeed, there are neurons where the influence of noise persists at high firing rates, such as in layer 5 of rat medial prefrontal cortex [24], however, the biophysical mechanisms that support this effect are absent in the standard LIF model.

In summary, population density methods (section: Population Density Approach) can replicate fluctuation-induced divisive gain modulation of the equilibrium response at low firing rates, previously observed in: simple integrate-and-fire models [14]–[16], simulations of biophysical realistic cell models [16],[20], as well as simulated conductance experiments *in vitro* [11].

### Divisive gain modulation with dynamic stimuli

**Figure 3:**
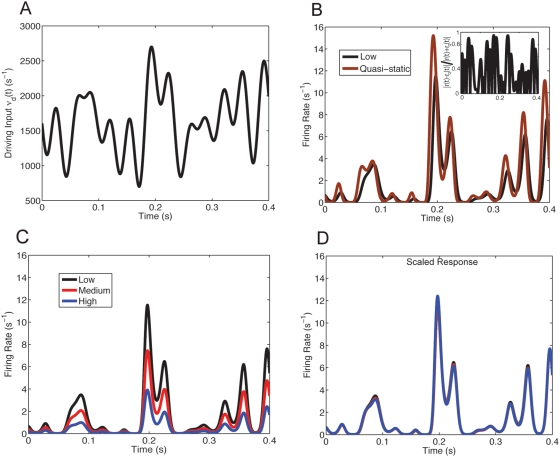
Gain modulation with temporally rich stimuli, and a refractory period. (A) The driving input 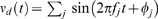 for 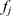 ranging from {1, 10, 25, 30, 50, 60}s^−1^ and 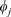 chosen randomly. (B) For the highly varying temporal driving input 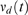 in (A) the output firing rate 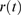 for balanced background synaptic input *low* computed using the population density method does not match the quasi-static approximation 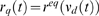 (brown curve). Inset: plot of 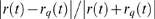 shows the absolute value of the difference of 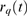 and the actual response 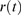 divided by the arithmetic mean can differ greatly. (C) The firing rate response to driving input in (A) with different balanced background levels. (D) The responses scaled with the same coefficients 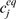 used in Figure 2C are used here (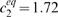 and 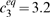).

We study the influence of background fluctuations on the nonequlibrium response to a highly time-varying excitatory drive. We choose an input rate 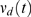 consisting of sums of sinusoids with various amplitudes, phases, and frequencies to mimic ‘rich’ time varying stimuli (for an example see Fig. 3A). This produces an inhomogeneous Poisson process driver input 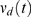, resulting in a non-stationary in time stochastic driving current. The response 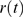 inherits the non-staionarities of 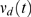 and is temporally modulated (Fig. 3B, black curve). Even though the stimulus results in a rather narrow range of response firing rates 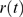, it has adequately rich temporal modulation to produce output firing rates that are different than the quasi-static response (Fig. 3B, brown curve), obtained by setting 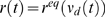.

The main result of our study is that fluctuation-induced divisive gain modulation is robust for low to moderate output firing rates in response to dynamic stimuli, despite the complicated dynamics of the leaky integrate-and-fire neuron in the nonequilibrium regime (i.e 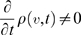). To demonstrate we compute the nonequilibrium responses (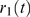, 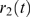, and 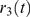), for the three levels of background activity used for the equilibrium case (low, medium, and high). For larger background activity the overall response is reduced, observed here since 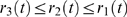 for all 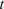 (Fig. 3C). We compute the dynamical analogue of 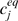, 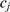 (see Equations (11) and (14)) and the scaled response 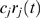, which quantitatively matches the base response 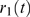 (Fig. 3D). This mimics the results for the equilibrium case (compare Figs. 2B and 3C and 3D). It is, a priori, unexpected that the dynamic response 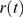 (with timescale 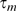 and refractory period 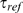) should scale in the same way as the equilibrium response 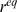.

**Figure 4:**
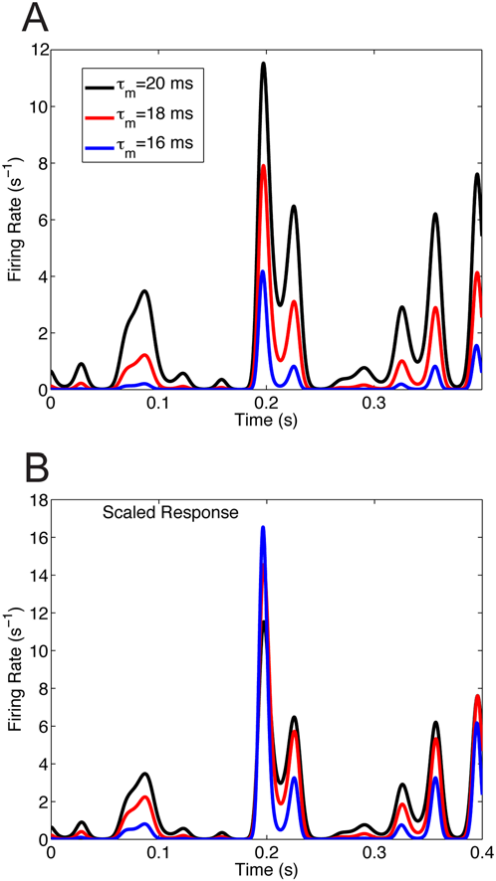
Background fluctuations required for divisive gain modulation. (A) The response with the same driver input as before (Fig. 3A), but with increased leakage conductance 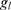 to mimic various background synaptic activity (conductance) levels in a deterministic way. The balanced background fluctuations levels (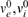)are the same (low) in all 3 curves. The black curve has 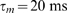 (same curve in Fig. 3), the red curve has 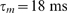, and the blue curve has 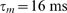. (B) The three responses do not scale in a simple manner. A least squares fit 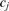 (Equation (14)) was used.

Previously, Holt & Koch [10] showed that an increase in membrane shunting without a change in input fluctuation causes a translational shift, rather than division of the equilibrium response curves, which was also verified by Chance et al. [11]. To verify that a pure shunting change cannot result in divisive gain modulation of the nonequilibrium responses (Fig. 3D), we fix the background fluctuation level and driver 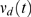, but increase the deterministic leakage conductance 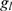 (Equation 1) to mimic different background synaptic activity (conductance) levels. Equivalently, 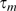 is replaced with a scaled version: 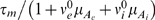, which has the same mean conductance in the absence of driving input as (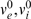). In simulations where the background activity is set by deterministic leak rather than by synaptic conductance fluctuations, the neurons had negligible firing rates because they were unlikely to fire by random chance and did not scale in a divisive manner (not shown). For exposition, we set all of the background fluctuation levels to that of *low* and vary 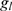, and hence 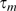, to mimic deterministic effects of changing background activity so that there are less fluctuations, but still some amount to induce background firing. The unscaled responses (Fig. 4A) were scaled via a least squares fit (Equation (14)). Not surprisingly, the responses do not scale in a divisive manner (Fig. 4B). Thus, divisive gain modulation in the nonequilibrium regime critically depends on changing the background fluctuation levels. We remark that Chance et al. were in the high conductance state when they verified this whereas our regime has less overall conductance.

**Figure 5:**
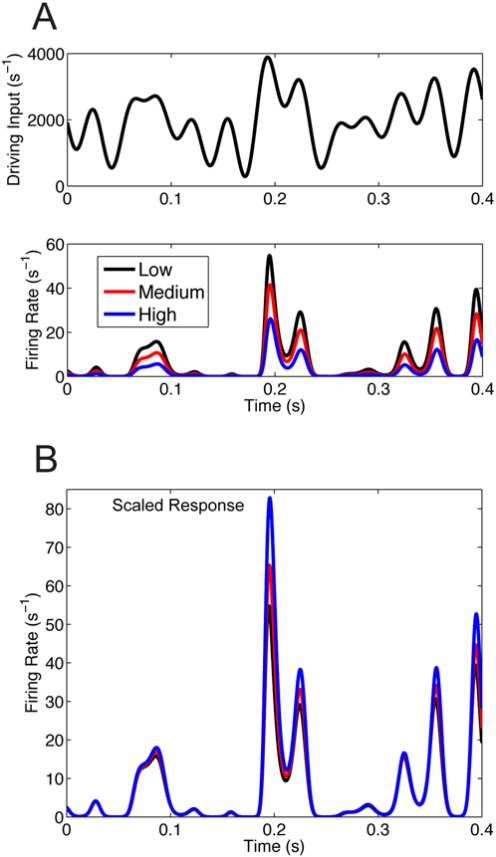
Divisive gain modulation does not hold for high intensity input drivers. (A) Top Panel: Same stimulus in Figure 3, but magnified to include higher output firing rates. Bottom Panel: The response for different balanced background levels 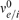. (B) The three responses do not scale in a simple way.

When the dynamic stimuli are increased so that resulting output firing rates are larger, the neurons no longer exhibit divisive gain modulation. Increasing the overall intensity of the driving input 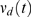 (compare Fig. 5A with Fig. 3A) yields firing rates 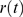 that are an order of magnitude larger (compare Fig. 5B with Fig. 3C). Increasing the overall background activity reduces the overall response magnitude (Fig. 5B), similar to what is observed in both the equilibrium and nonequlibrium regimes. However, when the response curves are scaled by 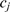 computed for the low rate case pure divisive gain modulation is not observed for the high rate response. There is no trivial (a time independent 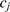) or natural way to scale the output firing rate curves so that they lie on top of each other. Since divisive gain modulation does not hold in the equilibrium setting for high output firing rates (drift dominated regime), one would expect that it does not hold in the nonequilibrium state. However, both the equilibrium and nonequilibrium states are quite different and we present the failure of fluctuation induced division for the sake of completeness. It is interesting to note that for periods of time when the output firing rates are low, divisive gain modulation appears evident, likely owing to a transient excursion into the fluctuation driven regime.

**Figure 6:**
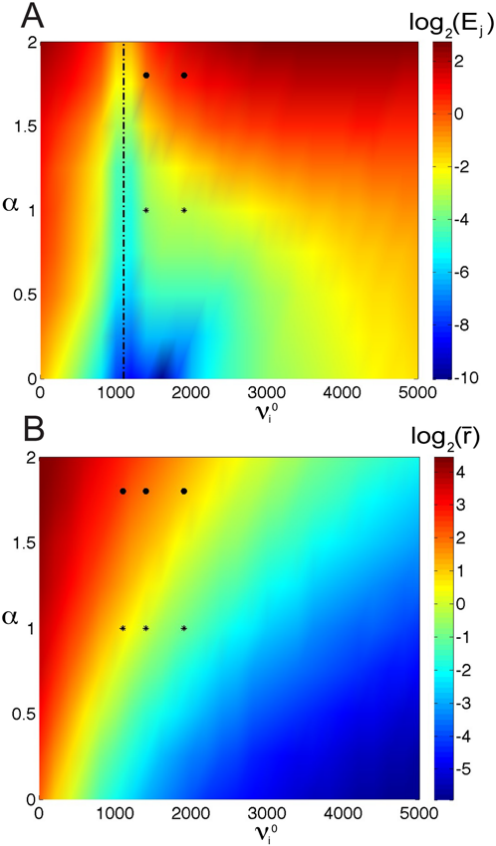
Parameter space where divisive gain modulation extends to the nonequilibrium regime. (A) The logarithm of the area (or error) between the time-dependent response curve scaled by the equilibrium scale factor 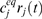 and the 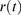 curve for many background levels and many driver inputs: 
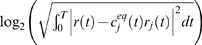
. The 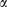 values on the vertical axis corresponds to a scaling of the driver input used in Figure 3A (see text for details). The two points at 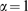 and 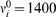, 1900 s^−1^ marked by stars (*) correspond to the difference in area between the curves in Figure 3D, and the two black circles (•) at 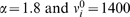, 1900 s^−1^ correspond to the difference in area between the curves in Figure 5B. Any region with colors in the range of orange to blue correspond to parameters where divisive gain modulation persists. (B) The average (unscaled) time-dependent response of the neurons with the same parameters and driver inputs as (A) are plotted on a logarithmic scale. The three stars (*) at 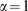 are the average firing rates of the unscaled responses in Figure 3C, and the three black circles (•) at 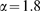 are the average firing rates of the unscaled responses in Figure 5A (bottom panel). A logarthmic scale was used to better highlight the variety of values.

In our model, when the driving input rate 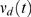 is low, the population of neurons rarely fire action potentials (i.e., low spontaneous activity). The firing rates in our simulations in this state range from nearly 0 to 3 s^−1^, depending on the background level of activity. Although extracellular recordings in the cortex suggest the neurons can fire spontaneously at rates larger than 2 s^−1^ [43], such experiments are usually biased towards active neurons. Extracellular recordings by [44] that were unbiased towards responsive neurons suggest that many neurons have low spontaneous firing rates and that only a small fraction of neurons respond ‘well’ to stimuli in unanesthetized animals; this fact was also discussed in [43]. Moreover, calcium imaging experiments of awake and anesthetized rats in layer 2/3 of the cortex show that many neurons have resting firing rates less than 1 s^−1^ [45]. The actual firing rate of neurons in the resting state is a contentious issue, but our results hold for many parameter regimes (see Fig. 6).

Divisive gain modulation with dynamic stimuli is robust in the subthreshold regime (Fig. 6). To illustrate this point, the response 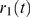 with *low* background level to a time-varying driver input and the response 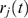 to the same driver input with a second level of background activity are computed. We plot the logarithm of the area (or error 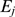, see Equation (13)) between the time-dependent response scaled by the equilibrium scale factor 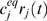 and the 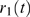 response:
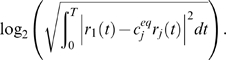
A logarthmic scale was used (Fig. 6) to better highlight the variety of values. The driver input is scaled as follows:
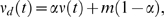
where 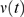 is the driver input used previously (see Fig 3A), 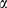 is the scaling parameter, and 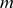 is a parameter that insures 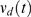 in positive and not too small. Notice 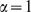 corresponds to the driver input in Fig. 3A, and 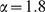 with 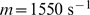 corresponds to the driver input in Fig. 5A. The vertical dot-dashed line in black corresponds to 0 error because it is the reference background curve for a given 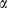. The two points marked by stars (*) in Fig. 6A at 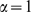 and 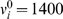, 1900 s^−1^ correspond to the difference in area between the curves in Fig. 3D, which is quite small. In fact, for a large region of parameter space, divisive gain modulation holds (any patch that is orange to blue in Fig. 6A). The two black circles (•) in Fig. 6A at 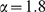 and 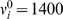, 1900 s^−1^ correspond to the difference in area between the curves in Fig. 5B. With larger 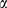 values, the neurons are in the drift dominated regime, and divisive gain modulation no longer holds, as expected (red regions in Fig. 6A).

The average (unscaled) time-dependent response of the neurons with the same parameters and driver inputs as Fig. 6A are plotted in Fig. 6B on a logarithmic scale:
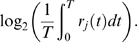
The three stars (*) at 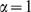 are the average firing rates of the unscaled responses in Fig. 3C, and the three black circles (•) at 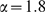 are the average firing rates of the unscaled responses in Fig. 5A (bottom panel). The average firing rate gives a qualitative idea of how large or small the response is as 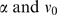 are varied. For example, the average firing rate at the low level is about 2 s^−1^ but the firing rate response can be quite low and as high as 15 s^−1^ (see Fig. 3B). Thus, divisive gain modulation holds for many parameters in a variety of subthreshold regimes.

### Comparison of optimal scaling factor of equilibrium and nonequlibrium responses

**Figure 7:**
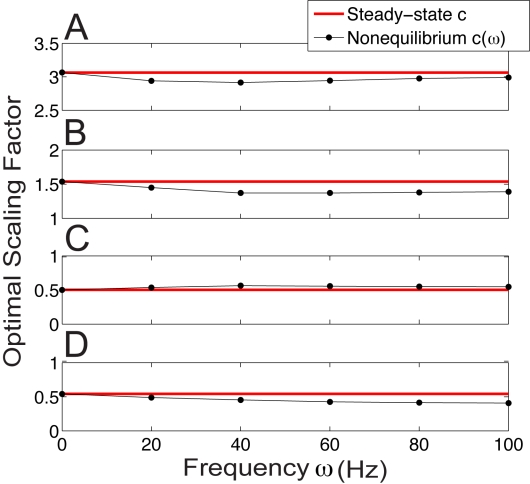
Optimal scaling factor for sinusoidal input compared with equlibrium scaling factor. The red line is the optimal scaling factor 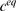 (11) of the equilibrium input/output curves computed for 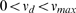, and the black line with dots is the optimal scaling factor 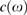 (14) of the two dynamical responses with sinusoidal input 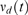. The scaling factors match very well for a variety of pairs of balanced background synaptic inputs. Top panels to bottom panels have background synaptic input rates of: (A) 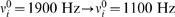, (B) 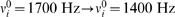, (C) 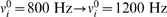, (D) 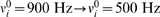. See Equation (4) for corresponding 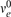 background balance of excitation and inhibition.

A gain control scheme will be effective in unpredictable environments if it is *quantitatively* insensitive to the timescales of the input, or in other words the degree to which the response is scaled should not depend on the spectral content of the signal. For fluctuation induced gain control we then require that the scaling factor 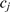 associated with a specific background state would need to be independent of the temporal frequencies in the driver input 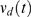. To test this we compare the optimal scaling factor 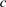 between two dynamical responses (each with a distinct balanced background state) where the synaptic driving input is:
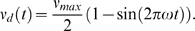
Notice the specified 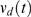 varies from 0 to 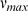, so that synaptic input rates are non-negative. Let us denote 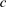 by 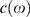 for two given background rates driven by sinusoidal input with frequency 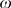 in Hz (here 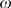 is not the conventional radian frequency). When 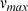 is in a low range the differences between 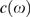 and 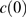 are negligible over a wide range of 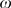 (Fig. 7). This result is robust for a range of background states (Fig. 7A–D). The quantitative match between the divisive scaling of equilibrium and nonequlibrium responses extends to more complicated temporal modulations of the driving input (Fig. 3A). Specifically, we find that 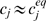 for the results shown previously (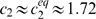 and 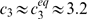 in Figs. 2 and 3). Thus, fluctuation induced gain control is quantitatively insensitive to the timescales of the driving input.

### Frequency response to weakly time varying inputs

To better describe the mechanism underlying fluctuation-induced divisive gain control in the nonequlibrium, we focus on a weak time modulation of the input drive and compute the linear frequency response [34],[46],[47]. The frequency response function gives the first order temporal modulation of output firing rate assuming the synaptic driving input consists of a large constant component and a small time-varying component:
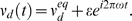
Here we have set 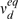 to be some fixed driver synaptic input rate, making the overall time independent excitatory input 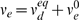, while the inhibitory input is still 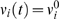. In total we then have the equilibrium state defined by the triplet 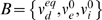, and the time dependent component of the driver simply 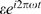. Assuming 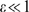 we approximate:
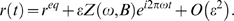
(15)The time modulation of the response is characterized by 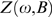, indicating how large or small the first order response is to time-varying input of frequency 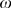 and amplitude 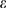. 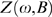 is a complex number with a modulus 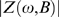 and phase 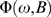: 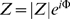.

**Figure 8:**
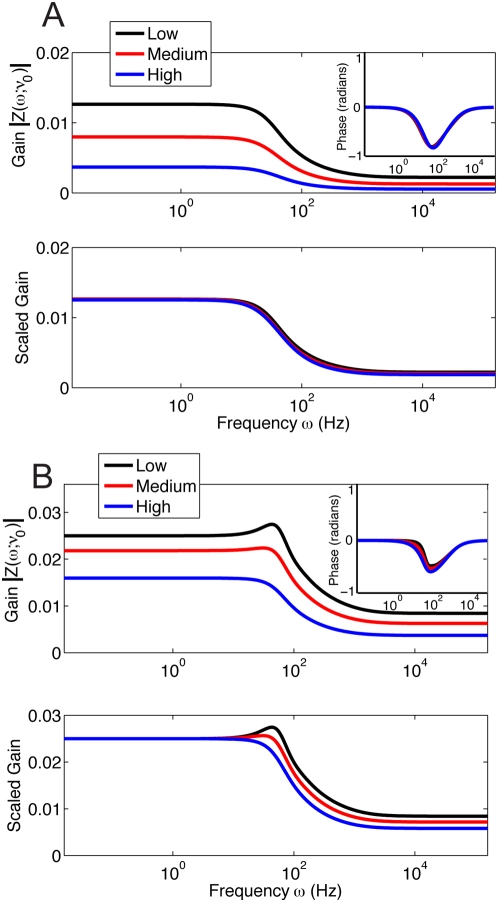
The frequency response in the fluctuation and drift dominated regimes. (A) Fluctuation driven regime. Top Panel: the frequency response is flat up to very large frequencies for all 3 different levels of balanced background activity. In all 3 cases, there was a constant excitatory 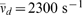 super-imposed on top of various balanced background inputs: *low*, *medium*, and *high*. Bottom Panel: the frequency responses for the 3 different levels of balanced background activity are scaled the same multiplicative factor (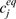 in Figs. 2 and 3) for a large range of frequencies. (B) Drift dominated regime. Top Panel: the frequency response for 3 levels of balanced background activity with a constant excitatory 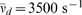 super-imposed on top of various balanced background inputs: low, medium and high. Bottom Panel: the frequency responses do not scale in a multiplicative manner.

Our earlier results (Fig. 3) show that for the same driver input 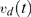 and different background inputs that 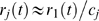 for some scaling factor 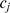. However, we know that in limit 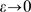 the equilibrium response also satisfies 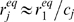 (Fig. 2). Combining these two results, and neglecting the 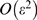 terms in Equation (15), predicts that 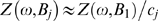 where 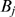 is a background state. Satisfyingly, when neurons are in the fluctuation-dominated regime 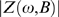 does indeed multiplicatively scale in the same quantitative manner for different levels of balanced background synaptic input scale (Fig. 8A and 8B). The phase component 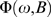 is the same for all 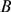 and 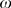 tested (insert Fig. 8A) and hence can not change the response 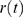 for different 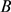. Thus from the quantitative scaling match of both 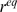 and 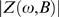 we expect fluctuation-induced divisive gain control to extend to weak inputs.

We remark that the near exact scaling of 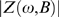 in the high frequency range (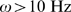) is important; if the multiplicative scaling was only true in the flat region of 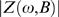 (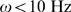) then fluctuation-induced divisive gain control would fail in the nonequlibrium, i.e. when the quasi-static approximation fails. To be more specific, 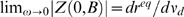, if we neglect the fluctuations given by the driver Poisson process. Thus the scaling of 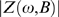 for 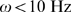 is completely explained by the scaling of the gain of 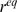. However, multiplicative scaling for 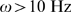 ensures that fluctuation induced gain control will extend into the nonequilibrium regime, even though the quasi-static approximation fails.

When the neurons are in the drift dominated regime, the frequency responses does not scale in a multiplicative manner because there are resonant peaks at integer multiples of the steady-state firing rate [34],[48],[49], and these resonant peaks occur at different frequencies for various balanced background synaptic activity (see Fig. 5B). Thus, divisive gain modulation with dynamic stimuli cannot possibly occur. The frequency responses in the drift dominated regime are scalar multiples of each other up to 10 Hz, where there appears to be divisive gain modulation with the same equilibrium scaling factors (see Fig. 5B). As explained in the previous paragraph, frequency response for 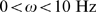 is equal to the frequency response for 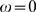. However, the multiplicative scaling breaks down for the same 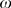 range where the quasi-static approximation breaks down, meaning that for drift dominated responses any fluctuation induced gain control in the equilibrium regime will not transfer to the nonequilibrium response.

## Discussion

Chance et al. [11] described a mechanism by which divisive gain modulation results from a balanced, fluctuating background synaptic activity which both shunts and linearizes the membrane to spike transfer. The response 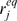 is a scaled version of a baseline condition 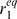, and the dividing factor 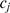 is independent of the driver intensity 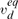. However, many stimuli induce input and output statistics which vary on the timescale of neural integration [26],[27],[30],[31]. Extending fluctuation induced gain control to accurately divide the response to these inputs is not automatic, as the spike-reset and refractory dynamics significantly shape the response in the nonequilibrium regime to be significantly different than the quasi-static approximation. However, our results show that the fluctuation induced gain control does extend to the nonequilibrium regime, increasing the potential utility of this form of gain control in neural processing. Furthermore, establishing the independence of the scaling term 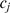 from the timescale of the driver greatly simplifies the circuitry required to implement gain control. In its simplest scenario, the gain of the response is set by the background rates 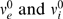 which maintain their scaling effect despite processing unpredictable environments where inputs statistics can vary dramatically.

The analysis of the time dependent response for weak signals showed how a scaling of 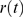 is inherited from an equivalent scaling of 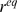 and the response function 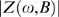 by fluctuating background conductances. The response to an input 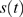 of arbitrary strength and spectrum can be written using the Volterra expansion [50]:
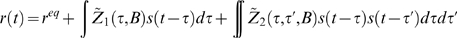


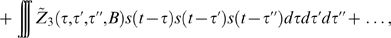
where 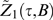 is the inverse Fourier transform of the response function 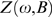 described in Equation (15). Fluctuation induced gain control extends well into the nonlinear regime, evidenced by the empirical agreement in regimes where 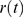 varies significantly about 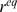 (Fig. 3D). In this case, the influence of the higher order terms in the Volterra expansion are likely important. We conjecture that, within the fluctuation dominated regime, each response function 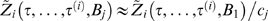, meaning that the multiplicative scaling extends, response function-by-response function, analogously into the nonlinear regime. This scenario is opposed to the one where each term exhibits scaling with distinct terms, yet the sum of terms somehow scales with 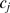, forcing agreement with our results where 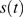 has large temporal variance (Fig. 3D). In principle computing 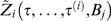 is quite difficult, however, if this scaling is correct then the influence of the stochastic background on 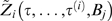, in the fluctuation driven regime, becomes straightforward.

Divisive gain control is a central tool in many neural computations [1], yet robust biophysical mechanisms that produce gain control are elusive [10]–[13],[16]. Our work gives further evidence that using background fluctuations as a mechanism to scale responses is a surprisingly stable mechanism operable for a variety of input statistics. Fluctuation induced effects on the equilibrium state transfer different from divisive gain control have been reported [24],[25]. Notably, [24] have shown that the firing response of pyramidal cells in layer 5 is sensitive to fluctuations at high rates, where the mean current no longer determines the spike rate. The mechanisms responsible are not present in the standard LIF model, however, modifications could possibly be made to model these effects and a population density equation could, in principle, be derived. These models would require more state variables and/or equations and in general are not computationally tractable without some reduction or approximation. Sophisticated methods for other neuron models have been developed [51]–[53]. Extending gain control to nonequilibrium responses to a larger class of models is currently an open avenue of research.

The LIF model we have used is an approximation to the dynamic clamp experiments of Chance et al. [11]. One difference is that our model does not have temporal correlations in the synaptic conductances, while there are temporal correlations in the experiments even though Chance et al. average over time (and trials) to obtain the firing rate. Also, we are using a simple yet biophysical spiking neuron model, where the level of background activity determines the variance of the background voltage (see Fig. 1B), consistent with the observations that membrane potential variability changes with the internal brain state [54]. In Chance et al. the variance of background voltage was the same for all background fluctuation levels, ensuring that the variability of the output firing rate is constant. Despite these differences, our results suggest that fluctuation induced divisive gain modulation is viable with dynamic stimuli.

The population density equations (6)–(8) that characterize the LIF model contain a partial differential-integral equation that is difficult to analyze. Our model is more general than white noise models that have an advection/diffusion density equation (e.g, Fokker-Planck equation) because it allows for large voltage changes upon receiving synaptic input events. However, the simulations shown in this paper are in the regime where the diffusion approximation is good. If the voltage change upon receiving synaptic events (excitatory or inhibitory) is assumed to be small, a good diffusion approximation of (6)–(8) is obtained by replacing 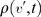 with 
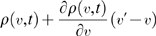
 in the integrals in the probability current terms 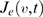 and 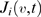 (see Text S1 and Figure S1). With large voltage changes, a similar approximation can be obtained by a re-scaling of the equation around the (deterministic) mean. However, a direct comparison to the Fokker-Planck equation with white noise conductances still must be done numerically because of the conductance-based input (Text S1 outlines the Fokker-Planck approximation to the full density equation and Figure S1 shows the magnitude of the advection/diffusion coefficients). Moreover, the analytic formulas obtained with advection/diffusion equations are often computed numerically and usually assume at least a quasi-static approximation. With Poisson current injection however, a closed form Fokker-Planck approximation is obtained with drift and diffusion coefficients that can be written exactly in terms of voltage, input rates, and the statistics of 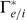. An analytical explanation of the robust scaling of the firing rate responses that is observed remains elusive yet is conceivable because of the many analytical results obtained for density equations of a variety of neuron models [51],[52]. However, we remark that even in the equilibrium regime an analytic explanation of divisive gain modulation via conductance fluctuations is difficult to obtain [14].

## Supporting Information

Figure S1 The advection/diffusion coefficients. (A) The functions d^e/i^_0_(v). (B) The functions d^e/i^_1_(v). Parameters: PSP = +0.5 or −0.5 mV (see main text for an explanation), τ_m_ = 20 ms, ε_i_ = −80 mV, ε_r_ = −70 mV, V_th_ = −55 mV, ε_e_ = 0 mV.
(8.28 MB TIF)

Text S1 Fokker-Planck approximation to full density equations
(0.01 MB TEX)
