# Correction: Divisive Gain Modulation with Dynamic Stimuli in Integrate-and-Fire Neurons

**DOI:** 10.1371/annotation/9c4474f6-e58e-41ea-af4b-090bbb86f474

**Published:** 2009-06-01

**Authors:** Cheng Ly, Brent Doiron

The Author Corrections are incomplete. The full Author Contributions are: Conceived of the study: CL BD. Performed the numerics: CL. Wrote the paper: CL BD.

