# Supplementary figures and images for: Divisive Gain Modulation with Dynamic Stimuli in Integrate-and-Fire Neurons
Source: PLoS Comput Biol. 2009 Apr 24;5(4):e1000365. doi: 10.1371/journal.pcbi.1000365 (PMC2667215; doi:10.1371/journal.pcbi.1000365)

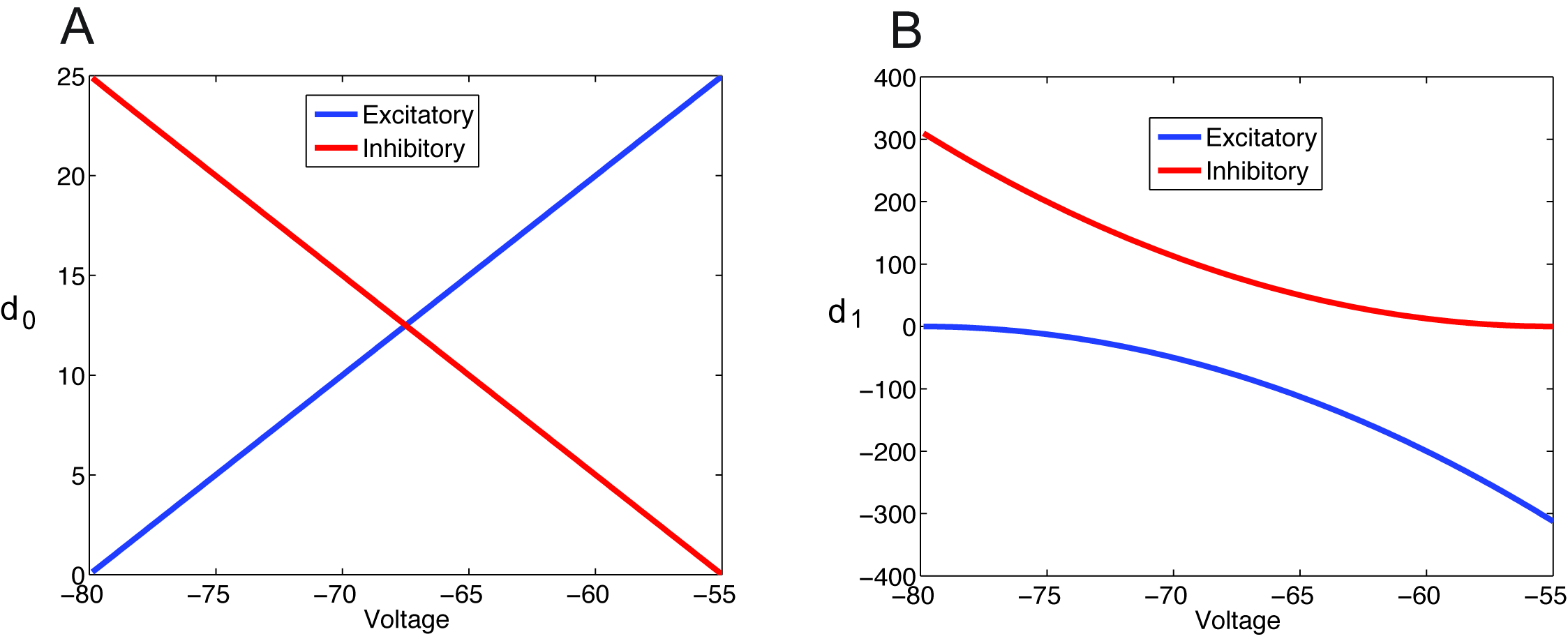

Supplement: Figure S1 — The advection/diffusion coefficients. (A) The functions de/i 0(v). (B) The functions de/i 1(v). Parameters: PSP = +0.5 or −0.5 mV (see main text for an explanation), τm = 20 ms, εi = −80 mV, εr = −70 mV, Vth = −55 mV, εe = 0 mV. (8.28 MB TIF) [file pcbi.1000365.s001.tif]
